# Magnesium biofortification of Italian ryegrass (*Lolium multiflorum* L.) via agronomy and breeding as a potential way to reduce grass tetany in grazing ruminants

**DOI:** 10.1007/s11104-019-04337-x

**Published:** 2019-12-02

**Authors:** Diriba B. Kumssa, J. Alan Lovatt, Neil S. Graham, Sarah Palmer, Rory Hayden, Lolita Wilson, Scott D. Young, R. Murray Lark, Beth Penrose, E. Louise Ander, Russell Thompson, Lin-Xi Jiang, Martin R. Broadley

**Affiliations:** 1 School of Biosciences, University of Nottingham, Sutton Bonington Campus, Leicestershire, UK; 2 Institute of Biological, Environmental and Rural Sciences (IBERS), Aberystwyth University, Aberystwyth, UK; 3 Tasmanian Institute of Agriculture, University of Tasmania, Hobart, Tasmania Australia; 4 Inorganic Geochemistry, Centre for Environmental Geochemistry, British Geological Survey, Keyworth, Nottinghamshire, UK; 5 Science and Advice for Scottish Agriculture, Edinburgh, UK

**Keywords:** Forage tetany index (FTI), Grass staggers, Hypomagnesaemia, Italian ryegrass, Magnesium chloride, Magnesium sulphate

## Abstract

**Aim:**

Magnesium (Mg) deficiency (known as grass tetany) is a serious metabolic disorder that affects grazing ruminants. We tested whether Mg-fertiliser can increase Mg concentration of Italian ryegrasses (*Lolium multiflorum* L.) including a cultivar (cv. Bb2067; ‘Magnet’), bred to accumulate larger concentrations of Mg.

**Methods:**

Under controlled environment (CE) conditions, three cultivars (cv. Bb2067, cv. Bb2068, cv. RvP) were grown in low-nutrient compost at six fertiliser rates (0–1500 μM MgCl_2_.6H_2_O). Under field conditions, the three cultivars in the CE condition and cv. Alamo were grown at two sites, and four rates of MgSO_4_ fertiliser application rates (0–200 kg ha^−1^ MgO). Multiple grass cuts were taken over two-years.

**Results:**

Grass Mg concentration increased with increasing Mg-fertiliser application rates in all cultivars and conditions. Under field conditions, cv. Bb2067 had 11–73% greater grass Mg concentration and smaller forage tetany index (FTI) than other cultivars across the Mg-fertiliser application rates, sites and cuts. Grass dry matter (DM) yield of cv. Bb2067 was significantly (*p* < 0.05) smaller than cv. Alamo. The effect of Mg-fertiliser rate on DM yield was not significant (*p* ≥ 0.05).

**Conclusions:**

Biofortification of grass with Mg through breeding and agronomy can improve the forage Mg concentration for grazing ruminants, even in high-growth spring grass conditions when hypomagnesaemia is most prevalent. Response to agronomic biofortification varied with cultivar, Mg-fertiliser rate, site and weather. The cost:benefit of these approaches and farmer acceptability, and the impact on cattle and sheep grazing on grasses biofortified with Mg requires further investigation.

**Electronic supplementary material:**

The online version of this article (10.1007/s11104-019-04337-x) contains supplementary material, which is available to authorized users.

## Introduction

Grazing landscapes and ruminant livestock have a dominant role in the environmental, economic and food security of many countries, especially in temperate regions. For example, in 2017/18, 72% of the UK’s land area (17.5 M ha) was utilised for agriculture; of that area the proportion of grazing land was 35% permanent grass, 22% sole rights rough grazing, 6.9% common land rough grazing, and 6.5% temporary grass (DEFRA 2018a). These grazing lands supported a ruminant livestock population of 10 million cattle and 35 million sheep. Among EU states, the UK has the largest sheep population and the third largest cattle population, after France and Germany (DEFRA 2018b). UK agriculture contributed £10.3 billion to the national economy in 2017 with grazing ruminants (i.e., cattle and sheep) representing >50% of this total (DEFRA 2018c).

Maintaining a thriving grazing ruminant sector requires supplying livestock with balanced nutrients through forages (Agricultural Research Council 1980; McDowell and Valle 2000; Voison 1963). Magnesium (Mg) is among the essential mineral nutrients for grazing ruminants, required to ensure a healthy skeleton, metabolism, cardiovascular and neuromuscular transmission (Ebel and Günther 1980; Schonewille 2013; Underwood and Suttle 1999). Livestock dietary Mg requirement vary depending on the species, breed, physiological conditions, age and growth rate of the animal, and the type of feed (Agricultural Research Council 1980). The recommended Mg concentration for grazing ruminants ranges between 1300 and 2200 mg kg^−1^ DM for cattle, and 900 and 1200 mg kg^−1^ DM for sheep (CSIRO 2007) with the critical recommended Mg concentration of 2000 mg kg^−1^ DM (Mayland and Hankins 2001; McDowell and Valle 2000).

In livestock, ~70% of Mg is stored in the skeleton and this pool is not easily mobilised when dietary Mg intake is reduced (Martens and Stumpff 2019; Suttle 2010). Hence, grazing ruminants need to be continuously supplied with forage that meets their Mg requirement. When the quantity of Mg supplied through feed is low, or when absorption in the rumen is impaired, the blood and cerebrospinal fluid Mg level can decline below clinical thresholds causing a physiological disorder known as hypomagnesaemic tetany (also known as grass tetany or grass staggers) (Dua and Care 1995; Henkens et al. 1973; Martens and Stumpff 2019; Suttle 2010). Ruminal absorption of Mg can be impaired by the imbalance of forage Mg^2+^, calcium (Ca^2+^) and potassium (K^+^) ions, which is termed as the forage tetany index (FTI) (Eq. 1) (Kemp and ‘T hart 1957). The risk of hypomagnesaemic tetany is considered to be high in livestock consuming forage with FTI exceeding 2.2 (Crawford et al. 1998; Elliot 2008; Kemp and ‘T hart 1957; Mayland and Hankins 2001; McNaught et al. 1973; Metson et al. 1966). Annually, in the UK hypomagnesaemic tetany is reported to affect 0.5% of dairy herds, and up to 10% on some dairy farms (Foster et al. 2007).

1 $$ \boldsymbol{Forage}\ \boldsymbol{Tetany}\ \boldsymbol{Index}\ \left(\boldsymbol{FTI}\right)=\frac{\boldsymbol{mEq}\ \boldsymbol{K}}{\boldsymbol{mEq}\ \left(\boldsymbol{Ca}+\boldsymbol{Mg}\right)} $$

*Where*, mEq is the milli equivalent of the elements (i.e., the elemental concentration (mg kg^−1^ DM) is divided by the atomic weight and multiplied by the valence of the respective elements).

In addition to cation imbalances in the forage, lush spring grass tends to be low in fibre and high in dry matter (DM) digestibility which accelerates rumen passage and reducing the ruminal absorption of Mg (Suttle 2010). This is further exacerbated by over application of K to grasslands as fertiliser, including from livestock manures (Agricultural Research Council 1980; Bhanugopan et al. 2015; Lunnan et al. 2018). Excess K^+^ in the soil solution suppresses the absorption of Mg^2+^ by plant roots (Elliot 2008).

There is considerable inter and intra-species variation in the concentration of Mg in forages. Mean concentration of Mg (mg kg^−1^ DM ± SD) reported include: grass hay 1400 ± 520, grass silage 1700 ± 540, clover silage 2300 ± 750, lucerne hay 1700 ± 270, maize silage 2200 ± 690 (Suttle 2010), and ryegrasses ranging between 1300 and 5500 (Crush 1983), 1100–6800 (Crush et al. 2018). Median Mg concentration in ryegrass and clover forages was reported to be 2200 (mg kg^−1^ DM) in New Zealand (Knowles and Grace 2014).

An Italian ryegrass (*Lolium multiflorum* L.) synthetic variety called Magnet (S417, Bb2067) with increased grass Mg concentration was bred by the Welsh Plant Breeding Station in the 1970s (Moseley and Baker 1991). This variety was shown to be effective in reducing hypomagnesaemic tetany in grazing sheep (Moseley and Baker 1991). However, the cultivar was never commercialised, due primarily to slightly smaller DM yield performance in National List trials in the late 1980s. The performance of cv. Bb2067 has not previously been assessed under altered Mg-fertiliser inputs. The aims of this research were (1) to test whether it is possible to raise the grass Mg concentration in Italian ryegrass by applying different rates of Mg-fertiliser, under controlled environment and field conditions; and (2) to explore the relative performance of cv. Bb2067 compared to modern Italian ryegrass cultivars under agronomic Mg biofortification.

## Materials and methods

### Controlled environment (CE) experiment

The CE experiment was conducted at the Sutton Bonington Campus of the University of Nottingham in 2016. Three cultivars, cv. Bb2067, cv. Bb2068 and cv. RvP were sown on 09 August 2016 in 576 cell trays (Plantpak plug tray 576 cell, Dejex Supplies Ltd., Donnington, UK) in compost (Levington F2 + S, Dejex Supplies Ltd) with a topping of fine horticultural grade silver sand (Dejex Supplies Ltd). The trays were transferred to a controlled environment room (20 °C day, 18 °C night, 16 h day length) and watered every other day with HortiMix standard (Dejex Supplies Ltd) at 10 ml L^−1^. After 21 days, plants were transplanted into 96 well trays (Dejex Supplies Ltd) containing low nutrient media (50% coarse sand, 50% Kettering loam, Dejex Supplies Ltd). Plants were watered every other day with Hortimix standard. In total there were 18 trays, each containing 8 plants of each line, randomly assigned to the central part of the tray. These plants were surrounded by cv. RvP plants to act as guard rows.

Thirty seven days after transplanting, the plants were cut leaving ~1 cm of aerial tissue. The cut plants were then watered every other day with 6 Mg treatments at 0, 75, 188, 375, 750 and 1500 μM MgCl_2_.6H_2_O. These were applied as 1 L per tray of a liquid feed based on Hoagland’s media, containing 0.25 m*M* KH_2_P0_4_, 0.5 m*M* KOH, 0.75 mM MgCl_2_.6H_2_O, 0.75 m*M* H_2_SO_4_, 0.1 m*M* FeNaEDTA, 2 mM Ca(NO_3_)_2_.4H_2_O, 2 m*M* NH_4_NO_3_, 30 μM H_3_BO_3_, 10 *μM* MnSO_4_.4H_2_O, 1 *μM* ZnSO_4_.7H_2_O, 3 *μM* CuSO_4_.5H_2_O, and 0.5 *μM* Na_2_MoO_4_.2H_2_O. The pH was maintained at 6.4.

All plants were sampled after 28 days, by cutting plants ~1 cm above the surface. The grass was placed in paper bags and oven dried at 50 °C until dry. The samples were digested in 2 mL 70% Trace Analysis Grade HNO_3_ and analysed by ICP-MS as described by Thomas et al. (2016).

### Field experiments design and treatments

Field experiments were conducted at Aberystwyth, Wales (52°26′00.6”N 4°00′36.7”W; 31 m.a.s.l.) and Edinburgh, Scotland (55°55′40.1”N 3°20′28.0”W; 57 m.a.s.l.) across two years (2017–2018). The soil type at Aberystwyth is well drained loam over gravel in the Eutric Endoskeleti-Eutric Cambisols (IUSS Working Group WRB 2007) Rheidol series (Cranfield University 2019). The site at Edinburgh has a coarse textured soil (sandy-silt-loam or sandy-clay-loam) in the Macmerry series (The James Hutton Institute 2019). A randomised complete block design was adopted with four and three replications at Aberystwyth and Edinburgh, respectively. Two treatment factors (cultivar and fertiliser rate), each with four levels provided 16 treatment combinations. The cultivars were cv. Bb2067, cv. Bb2068, cv. Alamo and cv. RvP. Cultivars Bb2067 and Bb2068 are large and small Mg accumulating cultivars, respectively, which have not been commercially released. Cultivars Bb2067 and Bb2068 were bulked progeny of 2 generations of selection for Mg content. Cultivar RvP was a current variety when these selections were made and individuals with large Mg selected from this cultivar comprised ~25% of cv. Bb2067 along with recurrent selections from the *Lolium multiflorum* breeding program. Cultivar Alamo is a modern and commonly grown commercial variety in the UK.

Plots of size 3 m * 1.2 m were sown in August 2016 at a seed rate of 35 kg ha^−1^. Compound NPK fertiliser (N [22%], P_2_O_5_ [4%], K_2_O [14%] SO_3_ [7.5%]) was added at a rate of 60 kg ha^−1^ prior to the first cut (March 2017), and then at 100, 100, and 60 kg ha^−1^ after cuts 1, 2, 3, respectively, and then 35 kg ha^−1^ after all subsequent cuts. No fertiliser was added after the final cut. The Mg fertiliser was applied in April 2017 and March 2018 as magnesium sulphate (MgSO_4_) at MgO equivalent rates of 0, 50, 100, and 200 kg ha^−1^. Reagent grade ≥ 97% anhydrous MgSO_4_ (Honeywell Specialty Chemicals GmbH, Seelze, Germany) was dissolved in warm water and applied with a calibrated knap sack sprayer after the first sward management cutting. Magnesium fertiliser application rates were scaled in relation to a recommendation of 50–200 kg ha^−1^ of MgO application every 3–4 years when exchangeable soil Mg is < 26 mg L^−1^ (AHDB 2017).

Grass harvesting was conducted using a Haldrup forage harvester at a cutting height of 5 cm above ground. In 2017, six grass cuts were taken at both sites. In 2018, five cuts were taken from Aberystwyth and seven from Edinburgh. Grass harvesting technique followed combined management as per UK National Lists trials protocol (Animal and Plant Health Agency 2019). During each cut, fresh weights were measured and DM yields were calculated after drying a 200–500 g subsample from each plot in a forced draught oven at 80 °C for 48 h. The dried sub-sample was milled, further subsampled, digested in 2 mL 70% Trace Analysis Grade HNO_3_ and analysed for concentration of Mg and other mineral elements by ICP-MS as described by Thomas et al. (2016), and certified reference materials were used to check analytical quality.

The FTI (Eq. 1) was calculated as the molar ratio of K^+^ to the sum of Ca^2+^ and Mg^2+^ in the grass. Where the FTI is >2.2, the risk of grass tetany in ruminants grazing on such feed is high (Kemp and ‘T hart 1957).

### Soil mineral composition analyses

Prior to sowing, composite soil samples (0–15 cm depth) were collected with an auger, using a “W” transect across each site to determine baseline soil physico-chemical properties. Soil samples were also collected from the 16 treatments (samples from the centre of each of four replicate plots were composited) at the beginning of June 2018, after the second Mg-fertiliser application. The baseline soil pH (in water), and exchangeable Mg, Ca and K were analysed at Lancrop Laboratory (Pocklington, UK) while the second-year soil pH, and exchangeable Mg, Ca and K concentrations were analysed at the University of Nottingham. At both laboratories, a similar procedure was followed. Thus, 5 mg of <2 mm sieved soil was dissolved in 25 mL of 1 *M* NH_4_NO_3._ The solution was shaken on an end-over-end shaker for 30 min followed by centrifuging for 15 min at 3000 rpm. The supernatant was then filtered using <0.22 μm syringe filter. The filtered solution was acidified with 0.2 mL of 50% (*v*/v) HNO_3_ and analysed using ICP-MS (ICP-MS; iCAP-Q, Thermo-Scientific, Loughborough, UK) at the University of Nottingham (Thomas et al. 2016), and inductively coupled plasma optical emission spectrometry (ICP-OES) at Lancrop.

### Data analysis

Data were compiled in MS Excel and Access. For field experiments where repeated observations (i.e., successive cuts) were made, statistical analyses were conducted using R (R Core Team 2018). Exploratory data analysis was undertaken on the residuals of an initial analysis of the data. The histogram of the residuals was inspected to assess the plausibility of an assumption of normality, and a plot of the residuals against the fitted values was inspected to assess the plausibility of an assumption that the variance of the residuals was homogeneous. When required, the data were transformed to natural logarithms to make this assumption plausible. Outliers were identified according to the outer fences of Tukey (1977) procedure whereby a datum is excluded if the residual lies more than three times the interquartile range below or above the first or third quartile, respectively. Accordingly, for grass Mg, FTI and S, the number of outlier data points excluded were two (Aberystwyth 2017), and three (Edinburgh 2018). In addition, for grass S, three more data points were excluded from Edinburgh 2018. A planned orthogonal set of contrasts was identified and mean comparison of grass Mg, FTI and S was conducted between, under field conditions, (i) the large Mg-accumulating cv. Bb2067 and other Italian ryegrass cultivars (ii) cv. Bb2068 and the two conventional varieties and (iii) cv. Alamo and cv. RvP. Under CE condition, comparison was made between i) cv. Bb2067 and the other two Italian ryegrass cultivars, and ii) cv. Bb2068 and cv. RvP.

Analyses of variances of the repeated observation (i.e., successive cuts) was addressed by the use of a linear mixed model. Two models were considered. In the first (sphericity assumption) where the correlation between the residuals for any two measures on the same unit were treated as uniform. In the second an exponential autocorrelation for successive measurements was assumed. The two models were fitted using the nlme package (Pinheiro et al. 2018) for the R platform (R Core Team 2018). The choice between the alternative models was then made based on Akaike’s information criterion (AIC), selecting the model for which this was smallest.

Analysis of variance on the grass dry matter yield and soil properties in the field experiments was conducted by fitting a generalised linear model without any transformation in MINITAB 18. Visualisations were also produced using MINITAB 18 (MINITAB 2017).

## Results

Raw mineral element concentration data of Italian ryegrasses of the CE experiment and field experiments, and exchangeable soil cations and dry matter yield of Italian ryegrasses data for the field experiments are given in Online Resources (Sup Table 1–4).

### Grass Mg concentration and Forage Tetany index (FTI) in the controlled environment (CE) experiment

**Fig. 1:**
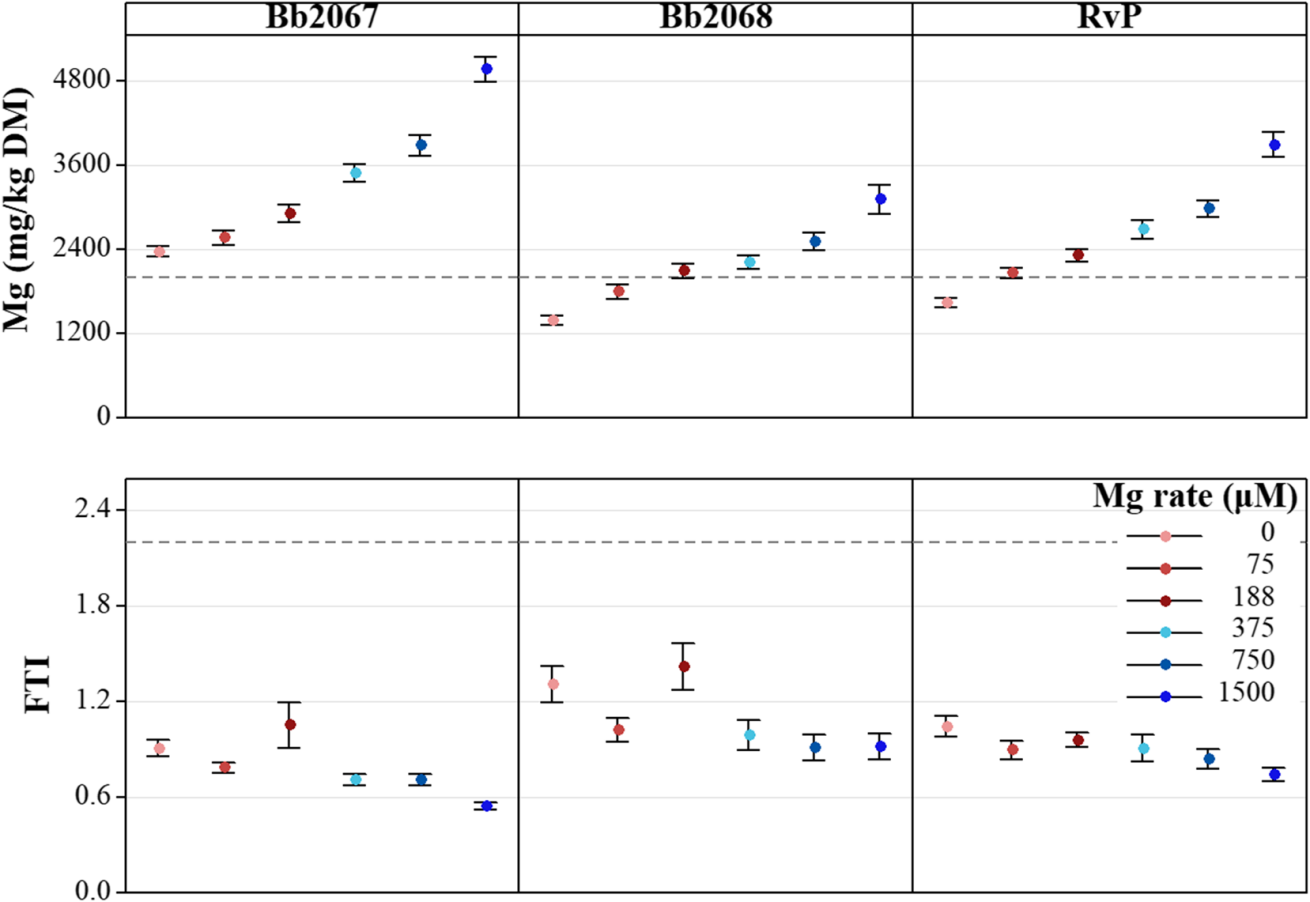
Mean grass Mg and Forage Tetany Index (FTI) of three Italian ryegrass cultivars (cv. Bb2067, cv. Bb2068, cv. RvP) grown under controlled environment conditions at six MgCl_2_.6H_2_O application rates (μM). Error bars are ± standard error (SE) of the mean, *n* = 24. Grey broken lines are the recommended (Mayland and Hankins 2001) minimum Mg concentration (2000 mg kg^−1^ DM) and the maximum for FTI (2.2) in forages (Kemp and ‘T hart 1957)

Grass Mg concentration increased with increasing Mg application rate (*p <* 0.05, Table 1), with all cultivars responding similarly (Fig. 1). Cultivar Bb2067 consistently had a greater Mg concentration than cv. RvP, which itself had consistently greater Mg than cv. Bb2068 at all Mg application rates (Fig. 1). Both planned contrasts among the cultivar means for Mg concentration were significant (*p* < 0.01) as shown in the Online Resource (Sup Table 5). At the largest Mg application rate (1500 μM MgCl_2_.6H_2_O), the grass Mg concentration (mg kg^−1^ DM; mean ± SD, *n* = 24) was 4966 ± 880 (cv. Bb2067), 3115 ± 1018 (cv. Bb2068), and 3889 ± 878 (cv. RvP). At the zero Mg application rate, the grass Mg concentration (mg kg^−1^ DM, mean ± SD, n = 24) was 2366 ± 381 (cv. Bb2067), 1382 ± 343 (cv. Bb2068), and 1629 ± 320 (cv. RvP). There was no significant interaction effect of cultivar × Mg application rate on grass Mg concentration (*p* ≥ 0.05) (Table 1).

**Table 1:** Analysis of variance of grass Mg concentration and Forage Tetany Index (FTI) in the controlled environment (CE) experiment, both after log-transformation. DF = degrees of freedom. *p* value = probability

Source	Numerator DF	Denominator DF	*p* value	
			Mg	FTI
Block	2	10	0.005	0.717
Mg application rate	5	10	<0.001	0.062
Cultivar	2	24	<0.001	<0.001
Mg application rate × cultivar	10	24	0.752	0.938

The FTI did not significantly (*p* ≥ 0.05) decrease with increasing Mg application rate (Table 1). Cultivar Bb2067 had the smallest FTI at all Mg treatment levels (Fig. 1). At the largest Mg application rate, the FTI (mean ± SD) was 0.55 ± 0.11 (cv. Bb2067), 0.92 ± 0.40 (cv. Bb2068), and 0.74 ± 0.22 (cv. RvP). At the smallest Mg-fertiliser application rate, the FTI (mean ± SD) was 0.91 ± 0.24 (cv. Bb2067), 1.31 ± 0.55 (cv. Bb2068), and 1.05 ± 0.32 (cv. RvP). As with Mg concentration, both orthogonal contrasts among the cultivar means were significant (*p* < 0.01) as shown in the Online Resource Sup Table 5. There was no significant interaction effect of cultivar × Mg application rate on FTI (*p* ≥ 0.05, Table 1).

### Soil properties at the field experimental sites

The baseline exchangeable soil Mg concentration was 86 mg L^−1^ at Aberystwyth and 193 mg L^−1^ at Edinburgh (Table 2). At both sites, exchangeable soil Mg concentration increased significantly (*p* < 0.05) due to the application of MgSO_4_ (Table 2). Thus, post-fertiliser application, the mean exchangeable soil Mg concentration (mg L^−1^) for control plots was 80 at Aberystwyth and 177 at Edinburgh. The increase in the exchangeable soil Mg concentration at Aberystwyth was 20%, 39%, and 74%, and at Edinburgh was 10%, 12% and 32%, at Mg-fertiliser application rates of 50, 100 and 200 kg MgO ha^−1^, respectively. Baseline exchangeable soil Ca and K concentration, and soil pH were below the recommended optimal for forage cultivation at both sites (Table 2) (AHDB 2017) and were not significantly affected by the application of MgSO_4_.

**Table 2:** Mean exchangeable soil Mg, Ca, and K concentration (mg L^−1^), and soil pH after the second year MgSO_4_ application, and baseline soil properties of the field experiment sites at Aberystwyth and Edinburgh. Post-treatment soil samples were collected on 31 May 2018 and 01 June 2018 from Aberystwyth and Edinburgh, respectively. Means that do not share letters are significantly (*p* < 0.05) different. n = the number of observations from which the mean was calculated. SD = standard deviation

Soil property	MgO equivalent application rate (kg ha^−1^)	Experimental site			
		Aberystwyth		Edinburgh	
		n	Mean ± SD	n	Mean ± SD
Mg	0	4	80.1 ± 6.4^d^	3	176.5 ± 8.8^c^
	50	4	96.4 ± 6.8^c^	3	193.5 ± 13.2^b^
	100	4	111.4 ± 8.5^b^	3	197.7 ± 2.9^b^
	200	4	139.2 ± 5.5^a^	3	233.3 ± 23.2^a^
Ca	0	4	1460. ± 123.3	3	1837 ± 162.2
	50	4	1364 ± 16.7	3	1703 ± 50.3
	100	4	1450 ± 114.6	3	1696 ± 136.4
	200	4	1365 ± 91.1	3	1703 ± 112.8
K	0	4	69.5 ± 7.8	3	35.5 ± 1.5
	50	4	60.7 ± 3.2	3	36.1 ± 4.5
	100	4	60.56 ± 5.53	3	40.1 ± 7.0
	200	4	58.30 ± 7.75	3	36.6 ± 2.8
pH	0	4	5.27 ± 0.03	3	5.3 ± 0.11
	50	4	5.25 ± 0.08	3	5.32 ± 0.04
	100	4	5.27 ± 0.05	3	5.36 ± 0.06
	200	4	5.33 ± 0.07	3	5.41 ± 0.04
Baseline					
Mg			86		193
Ca			1347		1713
K			85		73
pH			5.8		5.7

### Grass Mg and Forage Tetany index (FTI) in the field experiments

#### Aberystwyth

**Fig. 2:**
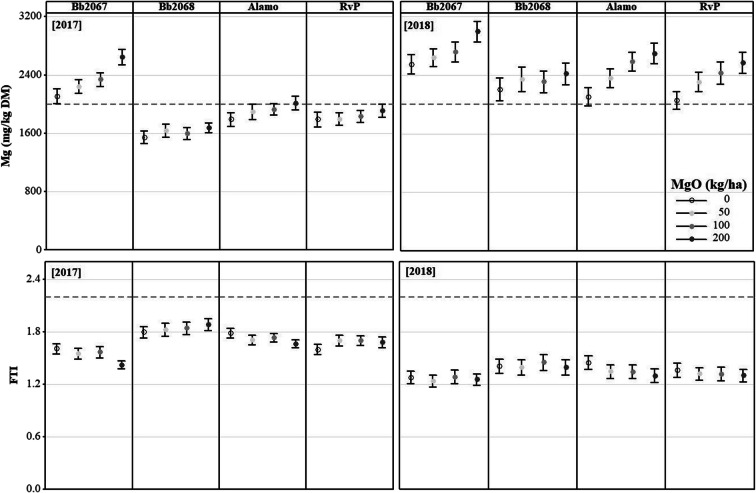
Mean grass Mg concentration, and Forage Tetany Index (FTI) of four Italian ryegrass cultivars (cv. Bb2067, cv. Bb2068, cv. Alamo and cv. RvP) at four Mg-fertiliser application rates (0–200 kg ha^−1^) in 2017 and 2018 , in the field experiment at Aberystwyth. Error bars are ±SE of the mean, n = 24 (2017), n = 20 (2018). Grey broken lines are recommended minimum grass Mg concentration (2000 mg kg^−1^ DM), and maximum for FTI (2.2)

**Fig. 3:**
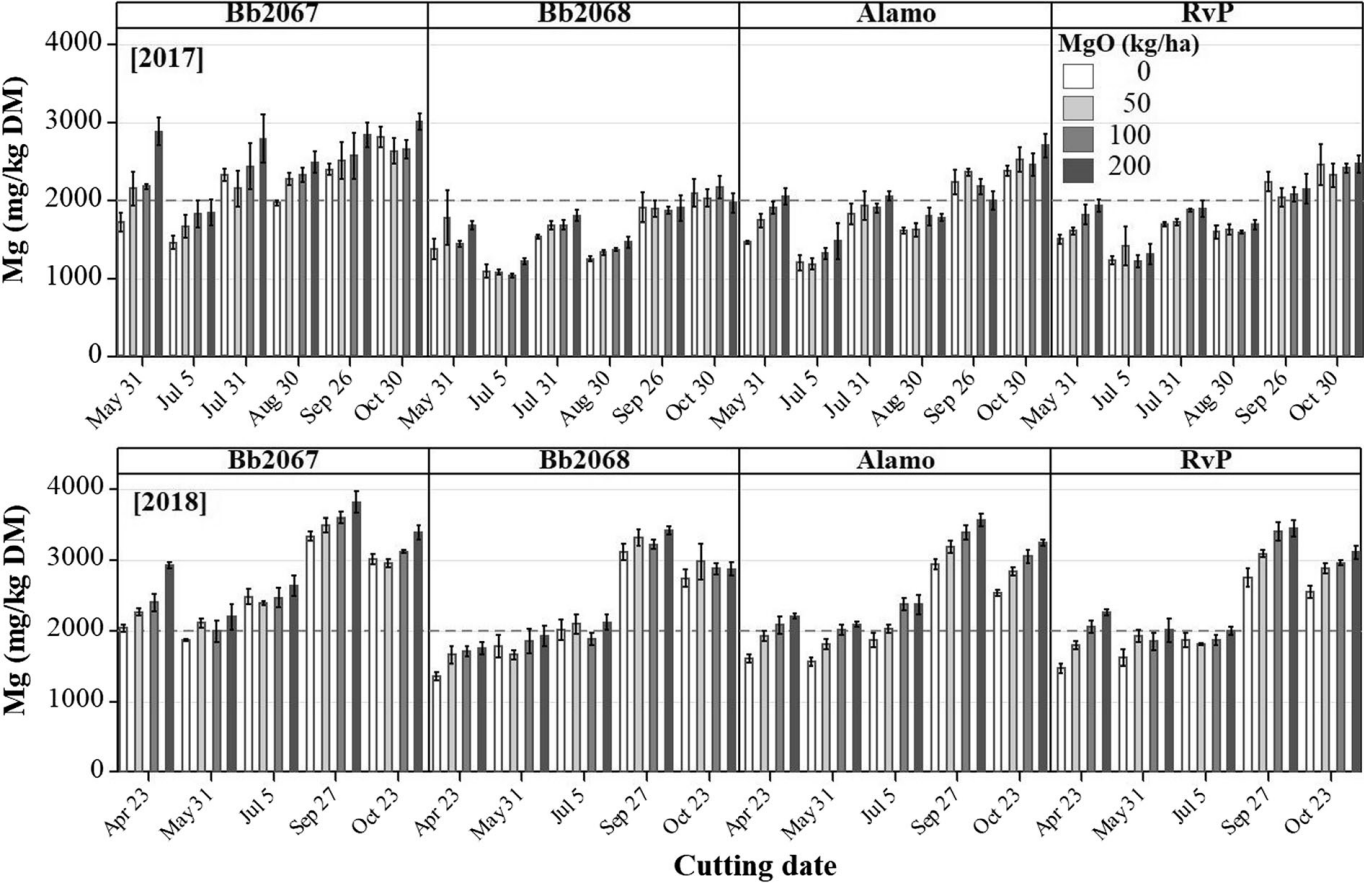
Mean grass Mg concentration of four Italian ryegrass cultivars (cv. Bb2067, cv. Bb2068, cv. Alamo and cv. RvP) at four Mg-fertiliser application rates (0–200 kg ha^−1^) at various cutting dates in 2017 and 2018 in the field experiment at Aberystwyth. Grey broken lines are recommended minimum grass Mg concentration (2000 mg kg^−1^ DM). Error bars are ±SE of the mean, *n* = 4

Grass Mg concentration increased with an increasing Mg-fertiliser application rate for all the cultivars in 2017 and 2018 (Fig. 2). Cultivar Bb2067 had greater grass Mg concentration than all other varieties at all Mg-fertiliser application rates, cuts and in both years (Figs. 2 and 3). Planned contrasts among the cultivar means for Mg concentration were significant (*p* < 0.01) except the one between cv. Alamo and cv. RvP as shown in the Online Resource (Sup Table 6). In 2017, at the largest Mg-fertiliser application rate (200 kg ha^−1^), the grass Mg concentrations (mg kg^−1^ DM; mean ± SD) were 2637 ± 506 (cv. Bb2067, *n* = 23), 1674 ± 314 (cv. Bb2068, *n* = 24), 2009 ± 454 (cv. Alamo, n = 23), and 1907 ± 431 (cv. RvP, n = 23). At the zero Mg-fertiliser application rate, the grass Mg concentrations (mg kg^−1^ DM; mean ± SD) were 2104 ± 495 (cv. Bb2067, n = 23), 1543 ± 423 (cv. Bb2068, n = 24), 1786 ± 457 (cv. Alamo, n = 24), and 1787 ± 490 (cv. RvP, n = 24). In 2018, at the largest Mg-fertiliser application rate, the grass Mg concentrations (mg kg^−1^ DM; mean ± SD, *n* = 20) were 2990 ± 628 (cv. Bb2067), 2414 ± 671 (cv. Bb2068), 2691 ± 624 (cv. Alamo), and 2563 ± 643 (cv. RvP). At the zero Mg-fertiliser application rate, the grass Mg concentrations (mg kg^−1^ DM; mean ± SD) were 2545 ± 589 (cv. Bb2067), 2201 ± 694 (cv. Bb2068), 2101 ± 572 (cv. Alamo) and 2050 ± 553 (cv. RVP) (Fig. 2). In 2018, there was a significant (*p* < 0.05) cultivar × Mg-fertiliser application rate, cultivar × cutting date, and Mg-fertiliser rate × cutting date interaction effect on grass Mg concentration. In 2017, there was cultivar × cutting date, and Mg-fertiliser rate × cutting date interaction effect on grass Mg concentration. There was no significant (*p* ≥ 0.05) cultivar × Mg-fertiliser application rate × cutting date interaction effect on the grass Mg concentration in either year (Table 3).

**Table 3:** Analysis of variance of grass Mg concentration and Forage Tetany Index (FTI) in the field experiment at Aberystwyth. DF = degrees of freedom. *p* value = probability

Source	Numerator DF		Denominator DF		p value			
					Mg		FTI	
	2017	2018	2017	2018	2017	2018	2017	2018
Block	3	3	45	45	0.014	0.030	<0.001	0.003
Cultivar	3	3	45	45	<0.001	<0.001	<0.001	<0.001
Mg-fertiliser rate	3	3	45	45	<0.001	<0.001	0.635	0.023
Cutting date	5	4	231	192	<0.001	<0.001	<0.001	<0.001
Cultivar × Mg-fertiliser rate	9	9	45	45	0.610	0.029	0.213	0.375
Cultivar × cutting date	15	12	231	192	0.003	<0.001	<0.001	<0.001
Mg-fertiliser rate × cutting date	15	12	231	192	<0.001	<0.001	0.153	0.950
Cultivar × Mg-fertiliser rate × cutting date	45	42	231	192	0.814	0.395	0.694	0.993

The FTI decreased with an increasing Mg-fertiliser application rate for all cultivars with cultivar Bb2067 having the smallest FTI. Planned contrasts among the cultivar means for FTI were significant (*p* < 0.01) except the one between cv. Alamo and cv. RvP as shown in the Online Resource (Sup Table 6). In 2017, at the largest Mg-fertiliser application rate the FTI (mean ± SD) was 1.42 ± 0.22 (cv. Bb2067), 1.88 ± 0.34 (cv. Bb2068), 1.66 ± 0.23 (cv. Alamo) and 1.68 ± 0.29 (cv. RvP). The FTI (mean ± SD) at the zero Mg-fertiliser rate was 1.60 ± 0.28 (cv. Bb2067), 1.88 ± 0.34 (cv. Bb2068) 1.78 ± 0.28 (cv. Alamo), and 1.60 ± 0.30 (cv. RvP) (Fig. 2). In 2018, at the largest Mg-fertiliser application rate, the FTI (mean ± SD) was 1.25 ± 0.28 (cv. Bb2067), 1.39 ± 0.40 (cv. Bb2068), 1.30 ± 0.35 (cv. Alamo) and 1.30 ± 0.33 (cv. RvP). At the zero Mg-fertiliser rate, the FTI (mean ± SD) was 1.28 ± 0.31 (cv. Bb2067), 1.40 ± 0.38 (cv. Bb2068), 1.45 ± 0.0.35 (cv. Alamo), and 1.36 ± 0.37 (cv. RvP) (Fig. 2). The FTI was significantly (*p* < 0.05) affected by cultivar, cutting date, and cultivar × cutting date interaction in 2017 and 2018, but the effect of Mg-fertiliser rate on FTI was only significant in 2018. There was no statistically significant (*p* ≥ 0.05) cultivar × Mg-fertiliser rate, Mg-fertiliser rate × cutting date, or cultivar × Mg-fertiliser rate × cutting date, interaction effect on the FTI (Table 3).

#### Edinburgh

**Fig. 4:**
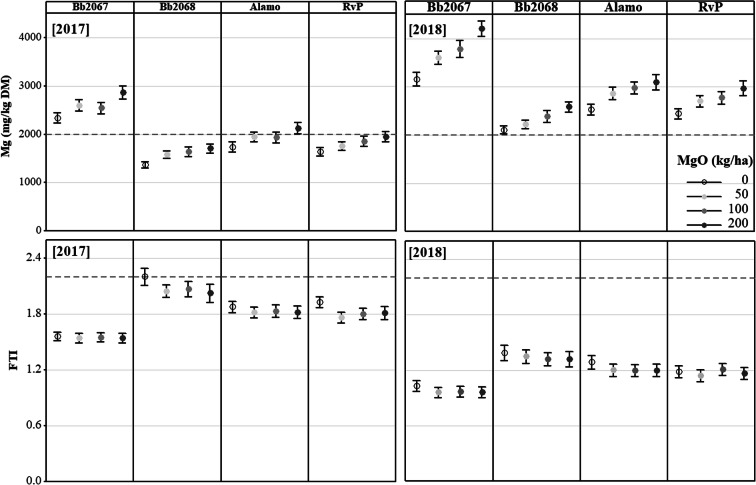
Mean grass Mg concentration, and Forage Tetany Index (FTI) of four Italian ryegrass cultivars (cv. Bb2067, cv. Bb2068, cv. Alamo and cv. RvP) at four Mg-fertiliser application rates (0–200 kg ha^−1^) in 2017 and 2018 in the field experiment at Edinburgh. Error bars are ±SE of the mean, n = 18 (2017), n = 21 (2018). Grey broken lines are recommended minimum grass Mg concentration (2000 mg kg^−1^ DM), and maximum for FTI (2.2)

**Fig. 5:**
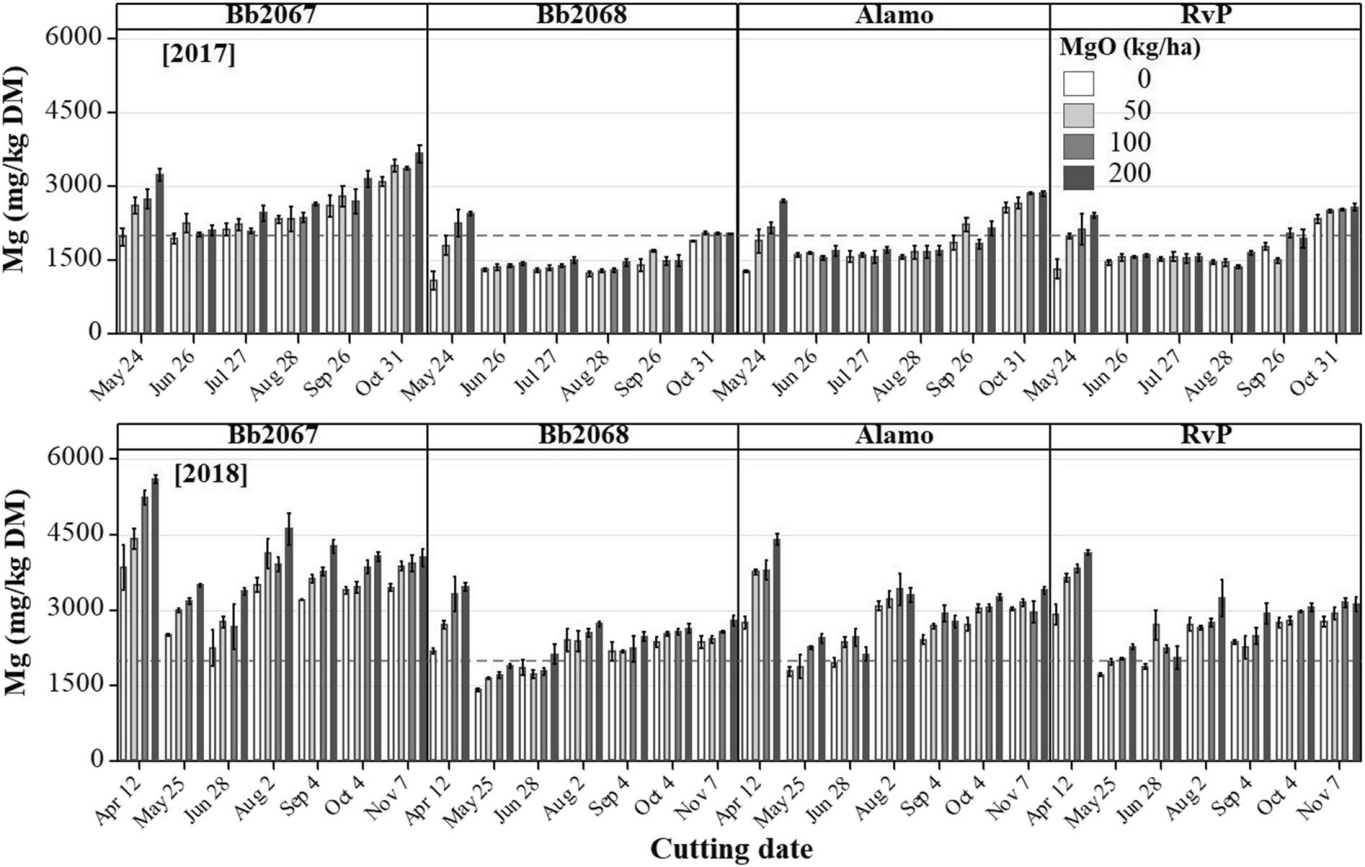
Mean grass Mg concentrations in four Italian ryegrass cultivars (cv. Bb2067, cv. Bb2068, cv. Alamo and cv. RvP) at four Mg-fertiliser application rates (0–200 kg ha^−1^) and at various cutting dates in 2017 and 2018 in the field experiment at Edinburgh. Grey broken lines are recommended minimum grass Mg concentration (2000 mg kg^−1^ DM). Error bars are ±SE of the mean, *n* = 3

Grass Mg concentration increased with an increasing Mg-fertiliser application rate for all cultivars in 2017 and 2018 (Fig. 4). Cultivar Bb2067 accumulated greater grass Mg concentration than cv. Bb2068, cv. Alamo and cv. RvP at all Mg-fertiliser application rates, cutting dates and in both years (Figs. 4 and 5). Planned contrasts among the cultivar means for Mg concentration were significant (*p* < 0.01) except the one between cv. Alamo and cv. RvP as shown in the Online Resource (Sup Table 6). In 2017, at the largest Mg-fertiliser application rate (200 kg ha^−1^), the grass Mg concentrations (mg kg^−1^ DM; mean ± SD) were 2801 ± 521 (cv. Bb2067, *n* = 17), 1698 ± 410 (cv. Bb2068, n = 17), 2122 ± 514 (cv. Alamo, *n* = 18) and 1945 ± 433 (cv. RvP, n = 18). At the zero Mg-fertiliser application rate the grass Mg concentrations (mg kg^−1^ DM; mean ± SD) were 2337 ± 478 (cv. Bb2067, n = 17), 1354 ± 304 (cv. Bb2068), 1732 ± 438 (cv. Alamo, n = 18) and 1632 ± 386 (cv. RVP, n = 17). In 2018, at the largest Mg-fertiliser application rate, the grass Mg concentrations (mg kg^−1^ DM; mean ± SD, *n* = 21) were 4205 ± 746 (cv. Bb2067), 2578 ± 503 (cv. Bb2068), 3094 ± 726 (cv. Alamo) and 2965 ± 707 (cv. RvP). At the zero Mg-fertiliser application rate, the grass Mg concentrations (mg kg^−1^ DM; mean ± SD, n = 21) were 3239 ± 531 (cv. Bb2067, *n* = 20), 2103 ± 395 (cv. Bb2068), 2526 ± 498 (cv. Alamo) and 2437 ± 480 (cv. RVP) (Fig. 4). In 2017 and 2018, the grass Mg concentration was significantly (*p* < 0.05) affected by cultivar, Mg-fertiliser application rate and cutting date. The cultivar × cutting date, and Mg-fertiliser application rate × cutting date interaction effect on grass Mg concentration was significant (*p* < 0.05) in both years. There was significant (*p <* 0.05) cultivar × Mg-fertiliser rate × cutting date interaction effect on grass Mg concentration in 2018 but not in 2017 (Table 4).

**Table 4:** Analysis of variance of grass Mg concentration and Forage Tetany Index (FTI) in the field experiment at Edinburgh. DF = degrees of freedom. *p* value = probability

Source	Numerator DF		Denominator DF		p value			
					Mg		FTI	
	2017	2018	2017	2018	2017	2018	2017	2018
Block	2	2	30	30	0.121	0.002	<0.001	<0.001
Cultivar	3	3	30	30	<0.001	<0.001	<0.001	<0.001
Mg-fertiliser rate	3	3	30	30	<0.001	<0.001	0.014	0.014
Cutting date	5	6	159	189	<0.001	<0.001	<0.001	<0.001
Cultivar × Mg-fertiliser rate	9	9	30	30	0.716	0.314	0.833	0.701
Cultivar × cutting date	15	18	159	189	<0.001	<0.001	<0.001	<0.001
Mg-fertiliser rate × cutting date	15	18	159	189	<0.001	<0.001	<0.001	0.037
Cultivar × Mg-fertiliser rate × cutting date	45	54	159	189	0.287	0.01	0.631	0.207

The FTI decreased with an increasing Mg-fertiliser application rate, for all cultivars, with cv. Bb2067 having the smallest FTI (Fig. 4). Planned contrasts among the cultivar means for FTI were significant (*p* < 0.01) except the one between cv. Alamo and cv. RvP in 2017 as shown in the Online Resource (Sup Table 6). In 2017, at the largest Mg-fertiliser application rate, the FTI (mean ± SD) was 1.53 ± 0.23 (cv. Bb2067), 2.02 ± 0.41 (cv. Bb2068), 1.82 ± 0.29 (cv. Alamo) and 1.81 ± 0.30 (cv. RvP). At the zero Mg-fertiliser application rate, the FTI (mean ± SD) was 1.57 ± 0.21 (cv. Bb2067), 2.15 ± 0.34 (cv. Bb2068), 1.88 ± 0.26 (cv. Alamo) and 1.89 ± 0.20 (cv. RvP). In 2018, at the largest Mg-fertiliser application rate, the FTI (mean ± SD) was 0.96 ± 0.26 (cv. Bb2067), 1.32 ± 0.38 (cv. Bb2068), 1.20 ± 0.31 (cv. Alamo) and 1.16 ± 0.30 (cv. RvP). At the zero Mg-fertiliser application rate, the FTI (mean ± SD) was 1.03 ± 0.28 (cv. Bb2067), 1.39 ± 0.38 (cv. Bb2068), 1.28 ± 0.34 (cv. Alamo) and 1.18 ± 0.29 (cv. RvP). In 2017 and 2018, the FTI was significantly (*p* < 0.05) affected by cultivar, Mg-fertiliser application rate and cutting date. Cultivar × cutting date, and Mg-fertiliser rate × cutting date interaction effect was significant (*p* < 0.05) on FTI in 2017 and 2018. There was no significant (*p* ≥ 0.05) cultivar × Mg-fertiliser, and cultivar × Mg-fertiliser rate × cutting date interaction effect on FTI in both years (Table 4).

### Grass dry matter yield from field experiments

**Fig. 6:**
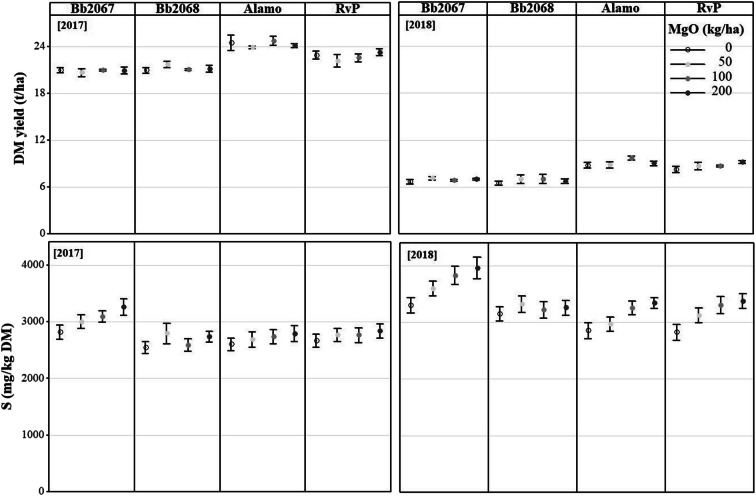
Annual total mean grass dry matter (DM) yield and mean grass S concentration of four Italian ryegrass cultivars (cv. Bb2067, cv. Bb2068, cv. Alamo and cv. RvP) at four Mg-fertiliser application rates (0–200 kg ha^−1^) in 2017 and 2018 in the field experiment at Aberystwyth. Error bars are ±SE of the mean, *n* = 24 (2017), n = 20 (2018)

The rate of Mg-fertiliser application did not significantly affect the annual total dry matter yield at either of the sites or in either year. At Aberystwyth, at the zero Mg-fertiliser application rate, total dry matter yields (t ha^−1^ p.a.; mean ± SD) were 21.0 ± 0.7 (cv. Bb2067), 21.0 ± 0.7 (cv. Bb2068), 24.5 ± 2 (cv. Alamo), and 22.9 ± 1.0 (cv. RvP) in 2017. At the largest Mg-fertiliser application rate (200 kg ha^−1^), total dry matter yields (t ha^−1^ p.a.; mean ± SD) were 21.0 ± 0.9 (cv. Bb2067), 21.2 ± 0.9 (cv. Bb2068), 24.1 ± 0.5 (cv. Alamo), and 23.3 ± 0.9 (cv. RvP). In 2018, at the zero Mg-fertiliser application rate, total dry matter yields (t ha^−1^ p.a.; mean ± SD) were 6.6 ± 0.6 (cv. Bb2067), 6.4 ± 0.5 (cv. Bb2068), 8.7 ± 0.7 (cv. Alamo), and 8.2 ± 0.8 (cv. RvP). At the largest Mg-fertiliser application rate, total dry matter yields (t ha^−1^; mean ± SD) were 6.9 ± 0.3 (cv. Bb2067), 6.7 ± 0.5 (cv. Bb2068), 9 ± 0.6 (cv. Alamo), and 9.1 ± 0.3 (cv. RvP) (Fig. 6).

**Fig. 7:**
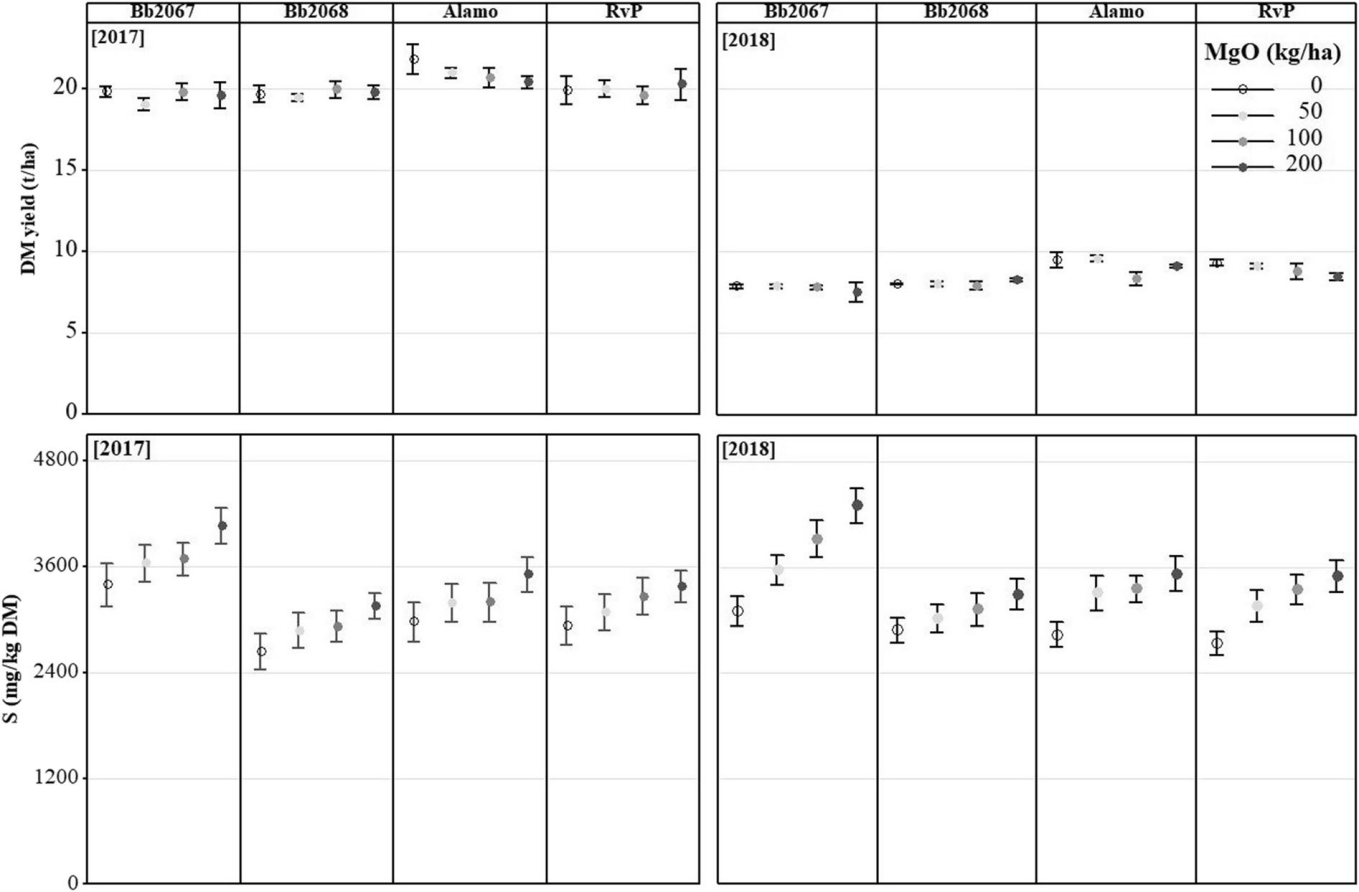
Annual total mean grass dry matter (DM) yield and mean grass S concentration of four Italian ryegrass cultivars (cv. Bb2067, cv. Bb2068, cv. Alamo and cv. RvP) at four Mg-fertiliser application rates (0–200 kg ha^−1^) in 2017 and 2018 in the field experiment at Edinburgh. Error bars are ±SE of the mean, n = 18 (2017), n = 21 (2018)

At Edinburgh, in 2017, at the zero Mg-fertiliser application rate, the total dry matter yields (t ha^−1^ p.a.; mean ± SD) were 19.8 ± 0.5 (cv. Bb2067), 19.6 ± 0.9 (cv. Bb2068), 21.8 ± 1.6 (cv. Alamo), and 19.9 ± 1.5 (cv. RvP). At the largest Mg-fertiliser application rate, total dry matter yields (t ha^−1^ p.a.; mean ± SD) were 19.6 ± 1.4 (cv. Bb2067), 19.7 ± 0.7 (cv. Bb2068), 20.4 ± 0.6 (cv. Alamo), and 20.2 ± 1.6 (cv. RvP). In 2018, at the zero Mg-fertiliser application rate, total dry matter yields (t ha^−1^ p.a.; mean ± SD) were 7.8 ± 0.2 (cv. Bb2067), 8 ± 0.1 (cv. Bb2068), 9.4 ± 0.8 (cv. Alamo), and 9.3 ± 0.3 (cv. RvP). At the largest Mg-fertiliser application rate, dry matter yields (t ha^−1^; mean ± SD) were 7.5 ± 1 (cv. Bb2067), 8.3 ± 0.2 (cv. Bb2068), 9.1 ± 0.1 (cv. Alamo), and 8.4 ± 0.4 (cv. RvP) (Fig. 7).

The total annual grass dry matter yield was affected by cultivar in both 2017 and 2018, with cv. Bb2067 and cv. Bb2068 having smaller yields than the commercial cultivars, cv. Alamo and cv. RvP. At both trial sites, the grass biomass yield in 2018 was 55–67% less than the yield in 2017 (Figs. 6 and 7) due to drought.

There were no significant (*p* ≥ 0.05) interaction effects of cultivar × Mg-fertiliser application rate, cultivar × cutting date, cultivar × Mg-fertiliser or cultivar × Mg-fertiliser rate × cutting date on the grass biomass yield at both sites in both years. There was a highly significant (*p* < 0.01) negative correlation between grass biomass yield and Mg concentration. The correlation between grass DM yield and Mg concentration at Aberystwyth was (in 2017, −0.308; in 2018, −0.495), and at Edinburgh was (in 2017, −0.194; in 2018, −0.447).

### Grass Sulphur (S) concentration from field experiments

At both field experimental sites, grass S concentration increased with an increasing Mg-fertiliser (MgSO_4_) application rate for all the cultivars in 2017 and 2018 (Figs. 6 and 7). Cultivar Bb2067 accumulated significantly (*p* < 0.05) more S in its biomass than the other three Italian ryegrass cultivars. Planned contrasts among the cultivars mean S concentration were significant (*p* < 0.01) except the one between cv. Alamo and cv. RvP as shown in the Online Resource (Sup Table 6). At Aberystwyth, grass S concentration was significantly (*p* < 0.05) affected by cultivar, Mg-fertiliser application rate, cutting date, and by the interaction effect of cultivar × cutting date and Mg-fertiliser rate × cutting date, in 2017 and 2018 (Table 5). There were no significant interaction effects of cultivar × Mg-fertiliser application rate × cutting date on grass S concentration (Table 5). At Edinburgh, in 2017 and 2018, the grass S concentration was significantly (*p* < 0.05) affected by cultivar, Mg-fertiliser application rate and cutting date. The cultivar × Mg-fertiliser application rate, and cultivar × cutting date interaction effect on grass S concentration was significant (*p* < 0.05) in 2018 but not in 2017 (Table 5). On the other hand, Mg-fertiliser rate × cutting date interaction effect on grass S was significant (*p* < 0.05) in both years. There was no significant (*p* ≥ 0.05) cultivar × Mg-fertiliser application rate × cutting date interaction effect on grass S concentration (Table 5).

**Table 5:** Analysis of variance of grass S concentration in the field experiment at Aberystwyth and Edinburgh. DF = degrees of freedom. *p* value = probability

Source	Numerator DF				Denominator DF				p value			
	Aberystwyth		Edinburgh		Aberystwyth		Edinburgh		Aberystwyth		Edinburgh	
	2017	2018	2017	2018	2017	2018	2017	2018	2017	2018	2017	2018
Block	3	3	2	2	45	45	30	30	0.314	0.210	0.806	0.792
Cultivar	3	3	3	3	45	45	30	30	<0.001	<0.001	<0.001	<0.001
Mg-fertiliser rate	3	3	3	3	45	45	30	30	<0.001	<0.001	<0.001	<0.001
Cutting date	5	4	5	6	231	191	155	192	<0.001	<0.001	<0.001	<0.001
Cultivar × Mg-fertiliser rate	9	9	9	9	45	45	30	30	0.252	<0.001	0.951	<0.001
Cultivar × cutting date	15	12	15	18	231	191	155	192	0.012	<0.001	0.651	0.011
Mg-fertiliser rate × cutting date	15	12	15	18	231	191	155	192	<0.001	<0.001	<0.001	<0.001
Cultivar × Mg-fertiliser rate × cutting date	45	36	45	54	231	191	155	192	0.344	0.003	0.737	0.293

At Aberystwyth, in 2017, at the largest Mg-fertiliser application rate (200 kg ha^−1^), the grass S concentrations (mg kg^−1^ DM; mean ± SD, n = 23) were 3257 ± 703 (cv. Bb2067), 2738 ± 476 (cv. Bb2068, n = 24), 2790 ± 697 (cv. Alamo) and 2838 ± 595 (cv. RvP). At the zero Mg-fertiliser application rate the grass S concentrations (mg kg^−1^ DM; mean ± SD, *n* = 24) were 2814 ± 588 (cv. Bb2067, *n* = 23), 2540 ± 512 (cv. Bb2068), 2599 ± 561 (cv. Alamo) and 2662 ± 573 (cv. RvP). In 2018, at the largest Mg-fertiliser application rate, the grass S concentrations (mg kg^−1^ DM; mean ± SD, *n* = 20) were 3970 ± 845 (cv. Bb2067), 3266 ± 592 (cv. Bb2068), 3345 ± 429 (cv. Alamo) and 3382 ± 604 (cv. RvP). At the zero Mg-fertiliser application rate, the grass S concentrations (mg kg^−1^ DM; mean ± SD, *n* = 20) were 3309 ± 601 (cv. Bb2067, n = 20), 3155 ± 575 (cv. Bb2068), 2861 ± 637 (cv. Alamo) and 2830 ± 639 (cv. RVP) (Fig. 6).

At Edinburgh, in 2017, at the largest Mg-fertiliser application rate (200 kg ha^−1^), the grass S concentrations (mg kg^−1^ DM; mean ± SD) were 4063 ± 844 (cv. Bb2067, *n* = 18), 3153 ± 600 (cv. Bb2068, *n* = 17), 3510 ± 860 (cv. Alamo, n = 18) and 3372 ± 782 (cv. RvP, n = 18). At the zero Mg-fertiliser application rate the grass S concentrations (mg kg^−1^ DM; mean ± SD, n = 17) were 3393 ± 1033 (cv. Bb2067, n = 18), 2638 ± 819 (cv. Bb2068), 2973 ± 898 (cv. Alamo, n = 18) and 2931 ± 904 (cv. RVP). In 2018, at the largest Mg-fertiliser application rate, the grass S concentrations (mg kg^−1^ DM; mean ± SD, *n* = 21) were 4303 ± 908 (cv. Bb2067), 3292 ± 813 (cv. Bb2068), 3526 ± 909 (cv. Alamo) and 3502 ± 820 (cv. RVP). At the zero Mg-fertiliser application rate, the grass S concentrations (mg kg^−1^ DM; mean ± SD, n = 21) were 3154 ± 747 (cv. Bb2067, n = 20), 2885 ± 645 (cv. Bb2068), 2835 ± 627 (cv. Alamo) and 2742 ± 614 (cv. RVP) (Fig. 7).

## Discussion

Raising grass Mg concentration to control hypomagnesaemic tetany in grazing ruminants is possible via agronomic and genetic biofortification of Italian ryegrass. Response to agronomic biofortification varied with cultivar, Mg-fertiliser rate, site and weather. Average grass Mg concentration in the large Mg-accumulator cv. Bb2067 was consistently above 2000 mg kg^−1^ DM forage Mg concentration threshold (Mayland and Hankins 2001) across sites and years when there was no Mg-fertiliser applied. Despite the contrasting extractable soil Mg at Aberystwyth and Edinburgh experimental sites, grass Mg concentration in the other Italian ryegrass cultivars was below 2000 mg kg^−1^ DM with or without Mg-fertiliser application in 2017. In 2018, the grass Mg concentration in all Italian ryegrass cultivars was well above the 2000 mg kg^−1^ DM threshold at both sites regardless of Mg fertiliser application. The contrast in grass Mg concentration between the two years is assumed to be due to the dilution effect of normal biomass production in 2017 compared with the smaller biomass yield in 2018 because of drought. The negative correlation between grass DM yield and Mg concentration also shows the biomass yield dilution effect on Mg concentration. Forage Ca, Mg and K imbalance was not observed in this study, as the average FTI of all Italian ryegrasses tested was <2.2 (Mayland and Hankins 2001) at all Mg-fertiliser levels under CE and field conditions. Nonetheless, cv. Bb2067 had significantly smaller FTI than the other cultivars.

### Agronomic biofortification of Italian ryegrasses with Mg fertilisers

Grass Mg concentration in Italian ryegrass can be increased by applying Mg-fertiliser. The current study used MgCl_2_ in the CE experiment, which is widely used source of Mg in liquid fertilisers (Mikkelsen 2010), and anhydrous magnesium sulphate (MgSO_4_) for the field experiment. There are a range of potential Mg resources including dolomitic limestone (for example, Bolan et al. 2005; McNaught et al. 1973; Mikkelsen 2010; Yermiyahu et al. 2017; Parnes 2013).

Under CE conditions, we observed 85–140% increases in grass Mg concentration across three Italian ryegrass cultivars at an application rate of 1500 μM MgCl_2_. In the field, there were 7–25% increases across the four Italian ryegrass cultivars at the application rate of 200 kg ha^−1^ MgO equivalent of MgSO_4_. In agreement with studies elsewhere (e.g., McNaught et al. (1973) the grass Mg concentration varied across the two experimental sites which had contrasting extractable soil Mg. Mg-fertiliser did not affect the dry matter yield of Italian ryegrass cultivars in either year, under adequate rain (2017) or drought (2018) conditions. Further steps should include cost-benefit analyses of using MgSO_4_ as a source of Mg-fertiliser to improve grass Mg concentration and its impact on the Mg status of ruminants grazing on the forages. Optimum frequency of Mg-fertiliser application and the responses of Italian ryegrass to different Mg-fertiliser types may also be worth exploring.

### Genetic biofortification of Italian ryegrasses with Mg is feasible

Cultivar Bb2067/Magnet was previously shown to reduce hypomagnesaemic tetany in grazing sheep (Moseley and Baker 1991). Here, Bb2067 had greater (5–73%) grass Mg concentration at all Mg-fertiliser application rates, cutting dates, and sites even when dry matter yields were affected by drought in 2018. Cultivar Bb2068 had the smallest concentration of grass Mg under corresponding scenarios. These two varieties were selected as large (cv. Bb2067) and small (cv. Bb2068) Mg-accumulators originating from the same group of commercial varieties through recurrent selection. Cultivar Bb2067 clearly shows the potential for genetic biofortification. However, it had not been commercialised due to its slightly smaller dry matter yield than Italian ryegrass cultivars of its time. The herbage dry matter yield of cv. Bb2067 compared with the largest yielding cv. Alamo over two years was 86% (2017) and 76% (2018) at Aberystwyth, and 93% (2017), 85% (2018) at Edinburgh. The average annual dry matter yield cv. Alamo was reported to be 18.06 t ha^−1^ DM (AHDB 2018). The average dry matter yield (> 20 t ha^−1^ DM) of cv. Bb2067 in these trials in 2017 was well above that perennial ryegrass under conservation sward management and comparable to cv. Alamo under farmer management. There is a need to transfer cv. Bb2067 Mg-accumulating trait into the cultivars that yield larger biomass (Capstaff and Miller 2018). To facilitate this process, it might be possible to identify genetic markers that are responsible for the accumulation of Mg in cv. Bb2067.

### Other potential nutritional consequences of Mg biofortification

There may be additional nutrient benefits for grazing ruminants given that MgSO_4_ application also increased grass S. Sulphur is an essential element for crops and livestock, and it is estimated that S deficiency is widespread in UK arable and pasturelands (Donald et al. 1999; Zhao and McGrath 1994) which could be mitigated by applying S-containing fertilisers. Here, there was no increase in DM yield due to MgSO_4_. Given the application of 7.5% SO_3_with NPK fertiliser and grass S concentration at the zero MgSO_4_ application rate was >2200 mg kg^−1^ DM (Suttle 2010), it seems unlikely that plants were affected by S deficiency. The application of MgSO_4_ to pastures can help to raise the sulphur:nitrogen (N) ratio in lush grasses with large N in spring during lambing season when animal requirement for S increases (Suttle 2010). Similarly, agronomic biofortification of forage with Mg-fertiliser can help the plants to readily take up Mg by raising the concentration of Mg^2+^ in the soil solution, curbing competition from other antagonistic ions such as K^+^ and NH_4_^−^, and dampening soil acidification effect on Mg^2+^ availability due to frequent application of inorganic or organic N-fertiliser to pastures (Bolan et al. 2005; Mulder 1956; Voison 1963). It will be important to quantify the wider effects of using fertilisers to improve grass nutritional quality in ruminant grazing systems. For example, it would be interesting to explore if dolomitic lime (with large concentrations of Mg) were to be utilised can increase grass Ca and Mg, and reduce the FTI and help to manage pasture soil pH in those areas where this is sub-optimal.

## Conclusions

This study has shown that biofortification of grass with Mg through breeding and agronomy can improve the forage Mg concentration in Italian ryegrasses for grazing ruminants, even in rapid-growth spring grass conditions when hypomagnesaemia is most prevalent. Response to agronomic biofortification varied with cultivar, Mg-fertiliser rate, site and weather. The Mg concentration in the grass biomass of the large Mg-accumulating cv. Bb2067 was greater while the FTI was smaller than the other three cultivars, at both field experimental sites. Cultivar Bb2067 consistently contained an average of >2000 mg kg^−1^ DM Mg at all Mg-fertiliser rates indicating its potential to reduce incidence of hypomagnesaemic tetany in grazing ruminants. Given, the slight DM yield penalty for growing cv. Bb2067 compared to cv. Alamo, transfer of the Mg-accumulating traits to the high DM yielder can be considered. The cost:benefit of these approaches, farmers’ adoption, and the impact of Mg-fortified Italian ryegrasses on cattle and sheep grazing on such grasses requires further investigation.

## Electronic supplementary material

ESM 1 (XLSX 45 kb)

ESM 2 (XLSX 149 kb)

ESM 3 (XLSX 14 kb)

ESM 4 (XLSX 12 kb)

ESM 5 (XLSX 9 kb)

ESM 6 (XLSX 12 kb)

## Abbreviations

AHDB: Agriculture and Horticulture Development Board
CE: Controlled Environment
DEFRA: Department for Environment Food & Rural Affairs
DM: Dry Matter
FTI: Forage Tetany Index
ICP-MS: Inductively Coupled Plasma Mass Spectrometry
MS: Microsoft
p.a.: Per annum
rpm: Revolutions per minute
SD: Standard Deviation
SE: Standard Error of the mean
